# Opium in Cerebro-Spinal Meningitis

**Published:** 1907-01-12

**Authors:** 


### OPIUM IN CEREBRO-SPINAL MENINGITIS.

Special interest attaches to an experience recorded by Dr. H. B. Currie, of Johannesburg, in the November issue of the *Transvaal Medical Journal*. The patient was a boy of ten years, in whom, on the third day of a febrile illness, mental dullness, retraction of the head, and Kernig's sign were recognised. Lumbar puncture obtained a milky fluid in which the specific micro-organism was found. On the eighth day a well-marked mottled rash appeared on the skin, and a favourable crisis was noted on the tenth day. Later, however, there was a return of the pyrexia, with severe headache and vomiting, but after some days these symptoms subsided, and the patient ultimately made a good recovery. In commenting on this clinical record, Dr. Currie remarks on the value of early lumbar puncture as an aid to diagnosis, and on the possibly favourable influence of the same agency in the scheme of treatment. But for the satisfactory termination of the case he gives the credit mainly to opium. This drug was given in large doses, and on the necessity for this Dr. Currie places the greatest emphasis. As much as one grain of morphine in association with atropine was given at a single dose, and in the course of 24 hours the patient received a total of not less than three grains of morphine, as well also as 10 minim doses of Battley's solution every six hours. The theory governing this procedure was that if the nervous system could be quieted down the responsible micro-organisms would in time develop sufficient toxin to check their further activity, and such a view can at least claim the justification of success. Repeated lumbar puncture and hot baths may have their value, but Dr. Currie gives a decided preference to opiates, provided these are given in full and repeated doses.

